# Graphene-supported Pd/Pt nano-catalysts for enhanced colorimetric detection of dopamine and NADH using paper-based microfluidic devices

**DOI:** 10.5599/admet.3247

**Published:** 2026-04-07

**Authors:** Ruri Agung Wahyuono, Jovin Jovin, Ignacius Gilbert Chano, Arda Fridua Putra, Annisa Septyana Ningrum, Muhammad Yusuf Hakim Widianto, Irkham Irkham, Yeni Wahyuni Hartati, Wulan Tri Wahyuni, Isnaini Rahmawati, Chi-Hsien Huang, Yi-Ting Lai

**Affiliations:** 1 Department of Engineering Physics, Institut Teknologi Sepuluh Nopember, Surabaya 60111, Indonesia; 2 School of Interdisciplinary Management and Technology, Institut Teknologi Sepuluh Nopember, Surabaya 60264, Indonesia; 3 Department of Mathematics, Institut Teknologi Sepuluh Nopember, Surabaya 60111, Indonesia; 4 Department of Chemistry, University of Padjadjaran, Sumedang 45363, Indonesia; 5 Department of Chemistry, Institut Pertanian Bogor (IPB) University, Bogor 16680, Indonesia; 6 Department of Chemistry, University of Indonesia, Depok 16424, Indonesia; 7 Department of Materials Engineering, Ming Chi University of Technology, New Taipei City 243303, Taiwan-

**Keywords:** Biomarker, colorimetry, nanocomposites, microfluidic paper-based analytical devices, sensor

## Abstract

**Background and purpose:**

Dopamine and nicotinamide adenine dinucleotide (NADH) are key biomarkers associated with neurological and metabolic disorders. Developing rapid, low-cost, and portable detection platforms of these biomarkers is essential for a point-of-care diagnostic kit. In this work, we report a colorimetric sensing approach using paper-based microfluidic devices (μPADs) modified with graphene-supported palladium (G/Pd) and platinum (G/Pt) nanocatalysts to enhance detection performance.

**Experimental approach:**

Monolayer G/Pd and G/Pt nanocomposites were synthesized via a hydrothermal method with precursor concentrations ranging from 0.1 to 10 mM. The catalytic behaviour and metal-graphene interactions were further investigated using spin-polarized density functional theory (DFT) calculation (PHASE/0). Microfluidic paper-based analytical devices (μPADs) were laser-printed on commercial filter paper and folded into 3D origami structures. Colorimetric responses were quantified using red, green, blue (RGB) and hue, saturation, value (HSV) analysis, where time-dependent Euclidean distance in RGB colour space was used to assess the reaction kinetics.

**Key results:**

DFT results indicate that Pd and Pt clusters preferentially adopt a top-site configuration on graphene, facilitating interfacial charge redistribution and enhancing catalytic activity experimentally. Catalyst-modified μPADs significantly improve reaction kinetics, reducing detection time by up to 3.7× for dopamine and 2.5× for NADH compared to unmodified devices. G/Pt (10 mM) exhibits the best overall performance, achieving limits of detection of 0.16 μM for dopamine and 0.195 μM for NADH with good linearity (*R*^2^ = 0.91). G/Pd displays competitive sensitivity, particularly at lower precursor concentration.

**Conclusion:**

The findings highlight that optimizing catalyst morphology and interfacial electronic structure is more critical than minimizing activation energy for achieving high-performance colorimetric sensing. The resulting platform shows potential as a cost-effective and portable tool for the detection of clinically relevant biomarkers in point-of-care settings.

## Introduction

Neurotransmitters such as dopamine play a crucial role as biochemical indicators of neurological function, alongside other important biomarkers, including uric acid, cholesterol, blood glucose, and nicotinamide adenine dinucleotide (NADH). Imbalances in neurotransmitters, particularly dopamine deficiency, have been linked to neurological disorders such as schizophrenia, depression, and Parkinson’s disease [1,2]. Clinical symptoms of Parkinson’s typically emerge after a 70 to 80 % loss of dopamine neurons [3]. In parallel, NADH, a key metabolic coenzyme involved in cellular redox reactions, has been increasingly recognized as a biomarker associated with neurological and metabolic disorders. Alteration in brain NADH levels is strongly associated with the progression of neurodegenerative diseases, including Alzheimer’s disease, where dementia is a primary symptom. Despite the known correlation between neurotransmitter concentration and mental disorders, early-stage detection remains limited, especially for Alzheimer’s disease, which is definitively diagnosed only postmortem [4].

Detection of neurotransmitters and metabolic biomarkers is typically performed using advanced techniques such as magnetic resonance imaging (MRI), electrochemical sensing, liquid chromatography, microdialysis, and various optical sensing methods, including spectroscopy and chemiluminescence [5-7]. However, these methods are often expensive and require sophisticated instrumentation, limiting their accessibility in developing and underdeveloped regions. Since 2007, paper-based microfluidic devices (μPADs) have emerged as promising low-cost, user-friendly alternatives for point-of-care diagnostics [8]. μPADs leverage the capillary action of paper substrates to direct fluid flow without the need for external power sources, and have been successfully applied in biology, chemistry, and medical diagnostics [9,10]. Their compatibility with the WHO’s ASSURED criteria (affordable, sensitive, specific, user-friendly, rapid, equipment-free and deliverable to end-users) makes them particularly appealing for resource-limited settings.

Microfluidic paper-based analytical devices (μPADs) primarily utilize optical or electrochemical detection methods [11]. For instance, Espinosa *et al.* [12] developed a chronoamperometric sensor for dopamine using Ag electrodes. Among these methods, colorimetric detection is especially attractive for μPAD applications due to its simplicity, enabling rapid visual identification *via* colour changes upon analyte-reagent interactions [13]. This method offers high sensitivity and selectivity for various analytes [14] and can be readily quantified using digital image processing tools [15]. To enhance analyte detectability, metal nanoparticles are typically employed as catalyst. For example, Zheng *et al.* [13] demonstrated that colour development on μPADs only occurred in the presence of catalytic Pt nanoparticles. Other nanocatalysts such as gold, silver, palladium, and bimetallic Au-Pd have also been used for sensitive peroxidase assays. Palladium, in particular, has shown promise for catalytic reactions in organic chemistry and biosensors due to its high activity [16,17]. These findings highlight the importance of integrating catalytic nanomaterials into biosensing platforms.

Further, graphene and its derivatives are widely used as support materials to enhance nanoparticle dispersion and prevent aggregation. For instance, Ragavan *et al.* [18] reported that graphene oxide-Pd composites exhibited a surface area 14 times greater than Pd nanoparticles alone. This high surface area helps maintain the catalytic activity of the nanoparticles [19]. Additionally, incorporating graphene into μPADs improves performance and reproducibility. Jia *et al.* [20] demonstrated that adding GO to glucose-detecting μPADs improved the uniformity of colour distribution, yielding more consistent results. Graphenemetal nanocomposites can be synthesized *via* physical or chemical methods. Physical synthesis methods, which involve mechanical forces or physical mixing, are cleaner but offer limited control over particle size and distribution [21]. In contrast, chemical methods allow for better control and integration. One common approach involves adding metal precursors to graphene oxide suspensions, followed by reduction to form the nanocomposite [22]. Hydrothermal synthesis, has gained popularity for fabricating graphene-metal nanocomposites, including combinations with gold [23], ruthenium [24], platinum [25], and palladium [26].

In this study, the catalytic performance of hydrothermally synthesized graphene-supported palladium (G/Pd) and platinum (G/Pt) nanocomposites is comparatively evaluated for the colorimetric detection of dopamine and NADH on μPAD platforms. The physicochemical properties of the nanocomposites and the modified μPADs are systematically characterized to assess their sensing performance. Both dynamic and static responses are analysed in relation to catalyst structure, providing insight into the interaction mechanisms between the nanocomposites and target analytes at the atomic level.

## Experimental

### Materials and reagent

The catalyst precursors used in this study included monolayer graphene from Ossila (UK) and hexachloroplatinic acid (H_2_PtCl_6_) and palladium chloride (PdCl_2_), both obtained from SmartLab (Indonesia). The hydrothermal synthesis used potassium iodide (KI) from Merck (Germany) and polyvinylpyrrolidone (PVP) K30 from BASF (Germany). Dopamine hydrochloride and grade II nicotinamide adenine dinucleotide hydrogen (NADH), both purchased from Merck (Germany), were utilized as the selected biomarkers. The reagents used for colorimetric detection included FeCl_3_, phenanthroline, 2,4-dinitrophenylhydrazine (DNP), potassium periodate (PPI), sodium hydroxide (NaOH), and resazurin salt, all obtained from Merck (Germany). Commercial-grade lab filter papers with a pore size of 15-20 μm and a thickness of 200 μm were obtained from a local supplier.

### Synthesis of graphene/palladium and graphene/platinum nanocomposite

Synthesis of monolayer graphene/palladium (G/Pd) and monolayer graphene/platinum (G/Pt) nanocomposite were carried out by the hydrothermal method following the method of Ragavan et al. with modification [18]. In this work, H_2_PtCl_6_ and PdCl_2_ concentrations were varied from 0.1 to 10 mM in 20 mL of distilled water and were mixed with KI in a mole’s ratio precursor to KI of 1:4. PVP was then added as the capping agent with the mass ratio of precursor to PVP of 1:115. Subsequently, 20 mL of monolayer graphene with a concentration of 0.2 mg/ml was added and mixed homogenously. The solution was then heated in a Teflon-lined autoclave at 180 °C for 90 minutes. Following the hydrothermal synthesis, the resulting suspension was centrifuged at 6000 rpm for 45 minutes, washed with ethanol, dried, and subsequently redispersed in 5 mL of distilled water. The synthesized G/Pd nanocomposites were designated as G/Pd (0.1), G/Pd (1.0) and G/Pd (10), corresponding to the varying concentrations of palladium precursors used in the synthesis. A similar naming convention was adopted for the G/Pt nanocomposites.

### Design and fabrication of 3D origami microfluidic paper-based analytical devices

**Figure 1.**
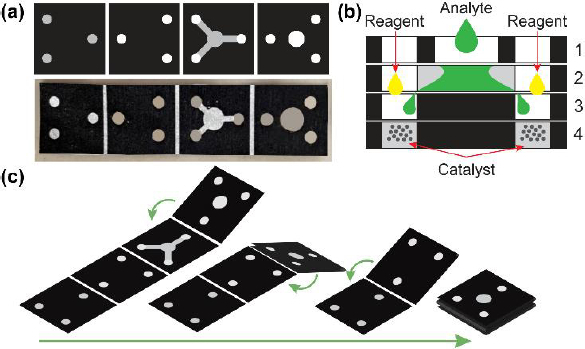
(a) designed and fabricated origami μPAD, (b) cross-section view of folded μPAD and (c) folding steps of origami μPAD

The structure of the μPAD depicted in Figure 1(a) consists of three detection zones, each 3 mm in diameter, along with fluid flow channels that are 2 mm wide and 4 mm long, and sample zones measuring 5 mm in diameter. The first layer is designed as the inlet channels into the next layers. The third layer is designed as an additional containment well to prevent liquid overflow from the μPAD. The analytes in the second layer will flow into the detection zone at the fourth layer and mix with the catalyst and reagent, thereby initiating the catalytic reaction and producing a colour change. The μPADs were created using commercial-grade filter paper, patterned with a laser jet printer (HP P1102), and subsequently heated in an oven at 155 °C for 12 minutes to establish hydrophobic barriers. Subsequently, the paper was punched and folded following Figure 1(c) to assemble the 3D Origami μPAD.

### Materials characterization

FTIR with a Thermo Nicolet iS50 was carried out to study the functional groups of Graphene, Pt, and Pd, as well as G/Pd and G/Pt nanocomposites, using infrared spectra. UV-vis absorption spectra of G/Pd and G/Pt were collected using a Thermo Scientific Genesys 150. FEI Inspect S50 Scanning Electron Microscope with Energy Dispersive X-Ray and HT7700 (Hitachi) Transmission Electron Microscope were used to study the surface morphology of G/Pd and G/Pt, and the images were further analysed with ImageJ software. X-Ray Photoelectron Spectroscopy data of G/Pd and G/Pt were performed using VG ESCA Scientific Theta Probe with a monochromated Al-Ka source. Atomic force microscopy using a Bruker Nanoscan and Contact Angle testing using an Ossila Goniometer were performed on the blank and modified μPAD to obtain surface topography and roughness.

### Computational method

First-principles calculations based on spin-polarized density functional theory were performed using the software PHASE/0 [27]. The exchange-correlation of the generalized gradient approximation was employed to treat the exchange-correlation functions, including van der Waals correction DFT-D3. The projected augmented wave (PAW) method was employed with a plane-wave energy cutoff of 5.449×10^-17^ J and a charge density cutoff of 4.905×10^-16^ J. The graphene matrix was modelled using a 4×4 supercell with a vacuum layer of 150 nm in the z-direction to avoid interlayer interactions. We focus on studying single atoms and small metal clusters (dimers (Pt_Top2 and Pd_Top2) and triangular trimers (Pt_Top3 and Pd_Top3) systems on the top of a graphene matrix. All atomic positions were fully relaxed until the atomic forces amounted to below 8.01×10^-8^ J m^-1^. A 10×10×1 Monkhorst-Pack k-point mesh was used for Brillouin zone sampling for supercell cases.

### Biomarker detection

Dopamine and NADH within a concentration range of 0.1 μM to 10 mM were used as the analytes to evaluate biomarker detection, with each biomarker dissolved in PBS at pH 7.4. Stock reagent solutions were prepared by dissolving 0.18 M FeCl_3_ and 14.5 mM phenanthroline in deionized water. Another reagent using resazurin was prepared by dissolving 0.8 mM resazurin salt in 0.1 M Tris-HCl buffer (pH 7.4). The μPAD was initially modified by depositing 2.5 μL of the G/Pd or G/Pt nanocomposite onto each detection zone. In the dynamic response evaluation to determine the optimal measurement time, the colorimetric reaction was performed directly by dropping 2.5 μL of the biomarker onto the detection zone in the fourth layer of the μPAD, thereby bypassing the origami concept. For dopamine detection, 2.5 μL of FeCl_3_ and phenanthroline was sequentially added to each detection zone. For NADH detection using resazurin, 2.5 μL of resazurin solution was added to each detection zone. Subsequently, the progression of the colour change reaction on the μPAD detection zones was recorded. Colorimetric responses were quantified using digital image processing tools to obtain RGB (red, green, blue) and HSV (hue, saturation, value) colour space values. The kinetics of the colorimetric reaction were assessed by quantifying the time-dependent colour change induced by the analyte, *i.e.* the Euclidean distance (*E*_d_) in RGB colour space, which was quantified over time using Equation (1):



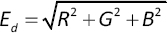

(1)


where *R*, *G* and *B* are intensities (0 to 255) for red, green and blue colour channels, respectively. This *E*_d_ represents the magnitude of the colour difference between two points in three-dimensional RGB space, with the reference point set to 0,0,0 (*R*, *G*, *B*). A calibration curve was then obtained using linear regression of the measured colour intensities for different dopamine and NADH concentrations. In this work, saturation and hue in the HSV colour space were used to construct calibration curves for dopamine and NADH, respectively. The performance of the μPAD was evaluated by analysing the calibration curve parameters, including linearity (*R*^2^), sensitivity (slope), limit of detection (LOD) and limit of quantification (LOQ), as described in our previous report [28].

## Results and discussion

### Physicochemical characteristics of G/Pd and G/Pt nanocomposites

**Figure 2.**
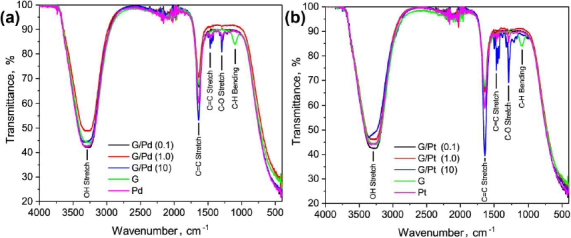
IR transmittance spectra of (a) G/Pd and (b) G/Pt nanocomposites with nanoparticle precursor concentration of 0.1, 1.0 and 10 mM

The molecular functional groups of G/Pd and G/Pt nanocomposites used as catalysts were evaluated from the IR transmittance spectra. As shown in Figure 2(a), the IR spectrum of the G/Pd nanocomposite exhibits a broad absorption band between 3735-2765 cm^-1^, attributed to O-H stretching vibrations from hydrated bonds, likely originating from distilled water used as the solvent. A sharp peak at 1636 cm^-1^ corresponds to C=C stretching vibrations in the carbon bonds of graphene, as confirmed by the reference graphene FTIR spectrum [29]. Additional peaks at 1465 cm^-1^ (C=C stretching) and 1290 cm^-1^ (C-O stretching) are attributed to the presence of polyvinylpyrrolidone (PVP) as a stabilizing agent during the G/Pd synthesis, as these peaks are absent in the pure graphene spectrum.

Absorption band at 1096 cm^-1^ appears only in the graphene spectrum and is not observed in Pt or G/Pt samples. This peak is associated with C-H bending vibrations, likely due to graphene dispersed in water. The observed increase in transmittance at this wavenumber suggests that the C-H bonds in graphene are broken, allowing the carbon to bond with platinum [30]. Another IR band at 556 cm^-1^ can be assigned to C-Cl stretching vibrations. Based on the presence of a peak at 1096 cm^-1^, it can be inferred that C-H bonds in the aqueous graphene solution are cleaved during synthesis, facilitating subsequent bonding with reduced platinum and chlorine species. A similar pattern is observed in the G/Pt nanocomposite spectrum shown in Figure 2(b). The G/Pt sample also used PVP as a stabilizer, as indicated by the presence of C=C and C-O functional groups. Moreover, the peak at 1096 cm^-1^ also appears in the G/Pt spectrum, supporting the interpretation that C-H bonds in graphene are broken during synthesis, thereby enabling bonding interactions with platinum in both nanocomposites.

To understand the electronic interaction between graphene and either palladium or platinum nanoparticles, computational results for G/Pt and G/Pd absorption spectra are discussed alongside the experimental absorption spectra. The optimized calculation of the lattice constant for graphene is found to be 0.248 nm and the C-C bond length is 0.143 nm, which is consistent with previous studies [31,32].

**Figure 3.**
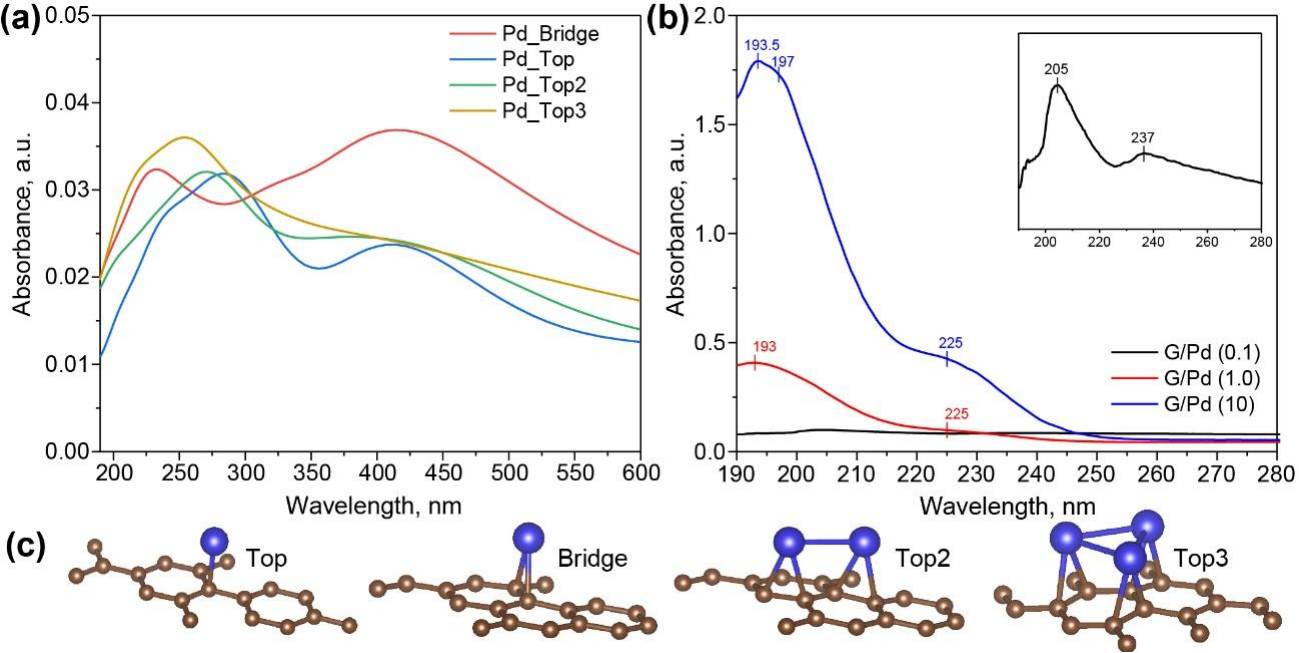
(a) UV-vis absorbance spectra of modelled G/Pd nanocomposite for various cluster configurations, (b) UV-vis absorbance spectra of synthesized G/Pd nanocomposite at different concentrations with the inset showing an enlarged absorbance spectrum of G/Pd (0.1), and (c) atomic configurations of Pd cluster models on graphene surface used for simulation (palladium atoms in blue and carbon atoms in brown)

**Figure 4.**
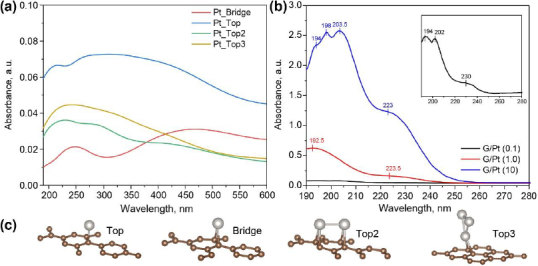
(a) UV-vis absorbance spectra of modelled G/Pt nanocomposite for various cluster configurations, (b) UV-vis absorbance spectra of synthesized G/Pt nanocomposite at different concentrations with the inset showing an enlarged absorbance spectrum of G/Pt (0.1), and (c) atomic configurations of Pt cluster models on graphene surface used for simulation (platinum atoms in light grey and carbon atoms in brown).

The bridge site (see Figure 3 and Figure 4), where a single atom of Pt or Pd is located above a C-C bond, is energetically lower than that on the top of the carbon matrix, with different energies being -1.088×10^-21^ J for G/Pt and -5.545×10^-23^ J for G/Pd. For a single atom, both Pt and Pd prefer absorption on high-symmetry sites (Pt_bridge and Pd_Bridge), where they induce minor distortion in the graphene lattice. The average bond-length distortion near a single Pt or Pd atom in the graphene matrix is found to be 0.145 nm. The bond length of C-Pt is 0.201 nm to 0.209 nm for Pt_Top and Pt_Bridge, respectively. In the case of G/Pd, the bond lengths of C-Pd indicate comparatively weaker interactions between a single Pd atom and the graphene matrix, where the C-Pd bond lengths are 0.208 nm and 0.218 nm for Pd_Top and Pd_Bridge, respectively.

The above-calculated bond lengths are consistent with Pd's slightly larger atomic radius and lower binding energy relative to Pt. When forming dimers (Pt_Top2 and Pd_Top2) on the graphene matrix, both systems adopt metal-metal bond lengths of 0.249 nm for Pt_Top2 and 0.273 nm for Pd_Top2. The metal atoms remain close to the graphene matrix, and the C-C bonding induces small local distortions in the hexagonal lattice. In the case of triangular clusters (Pt_Top3 and Pd_Top3), the structures tend to slightly deviate from an ideal equilateral triangle due to asymmetrical bonding with the carbon atoms of the graphene matrix, as shown in Figs. 3c and 4c. Two longer Pt-Pt bond lengths are found to be 0.249 nm, whilst the shortest is 0.244 nm. Pt_Top3 clusters tend to be closer to the graphene matrix with a Pt-C bond length of 0.213 nm, while Pd_Top3 clusters show a more relaxed geometry with slightly increased Pd-C (0.218 nm). In all cases, Pt atoms exhibit stronger hybridization with the graphene matrix's sp-orbitals, resulting in a more pronounced vertical bond and tighter binding than in the Pd cases. These geometric differences not only reflect the metal-graphene interactions but also affect the electronic and catalytic behavior of G/Pt and G/Pd systems.

We calculate binding energies (*E*_bind_) to evaluate the interaction strengths and stability of the systems. The *E*_bind_ was determined by subtracting the energy of pristine graphene matrix (*E*_G_) and the isolated metal atom or cluster (*E*_M_) from the total energy of the absorbed system (*E*_G/M_), which is calculated as follows:



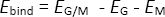

(2)


For single-atom adsorption, both metals preferentially occupy higher symmetry sites, with the bridge site being slightly more favourable than the top sites. The *E*_bind_ of a single Pt atom shows stronger adsorption than a Pd atom on both the top and bridge sites of the graphene matrix, as summarized in Table 1. The *E*_bind_ trend continues as cluster size increases: the Pt_Top2 on graphene matrix exhibits *E*_bind_ of -10.975 × 10^-19^ J, while the Pt_Top2 binds at -7.155 × 10^-19^ J. The Pt_Top3 binds more strongly compared to the Pd_Top3 system. The more negative binding energy of Pt across all configurations suggests stronger hybridization between Pt d-orbitals and graphene sp-orbitals, as well as greater binding energy within Pt clusters. These binding energy results indicate that Pt atoms form more stable, strongly bonded clusters on the graphene matrix than Pd atoms.

**Table 1.** Binding energies (*E*_bind_) of single Pt and Pd atoms and clusters on the top of the graphene matrix

System (Pd/Pt) configuration on graphene matrix	*E*_bind_ (×10^-19^ J)
Pt_Top	-4.931
Pt_Bridge	-5.227
Pt_Top2	-10.975
Pt_Top3	-10.807
Pd_Top	-3.911
Pd_Bridge	-3.926
Pd_Top2	-7.155
Pd_Top3	-10.847

Experimental absorption spectra of G/Pd nanocomposites depicted in Figure 3(b) show notable peaks around 200 nm and 230 nm at different Pd precursor concentrations. Compared with the modelled spectra in Figure 3(a), the spectrum of G/Pd (0.1) closely resembles the absorption profile of the Pd_Top configuration. In contrast, the spectra of G/Pd (1.0) and G/Pd (10) appear more similar to the Pd_Top2 configuration. A distinct feature of the G/Pd (0.1) sample is the presence of a shoulder preceding the main absorption band at 205 nm, which is absent in higher concentrations. Meanwhile, the broader shoulder around 230 nm observed in G/Pd (1.0) and G/Pd (10) may suggest the onset of inter-band transitions in palladium, which typically result in broad, less-defined absorption features due to electronic transitions from the d-band to the sp-band [33].

The UV-vis absorbance spectra of G/Pt nanocomposites at varying Pt precursor concentrations, as shown in Figure 4(b), exhibit a similar pattern to that observed for G/Pd. However, G/Pt exhibits additional distinct peaks between 190-200 nm, which may be attributed to higher-energy electronic transitions in platinum, potentially involving more complex inter-band processes than in palladium. Furthermore, a new absorption band around 225-240 nm can be attributed to the interaction between Pt nanoparticles and the π-electron system of graphene, which may lead to π-π* band splitting [34]. This suggests a degree of hybridization or electronic coupling between the metal and the graphene substrate. When compared to the modelled absorption spectra in Figure 4(a), the spectra of G/Pt (0.1) and G/Pt (10) most closely resemble those of the Pt_Top2, while G/Pt (1.0) resembles Pt_Top configurations, indicating the likelihood of similar coordination geometries between the Pt clusters and the graphene surface.

**Figure 5.**
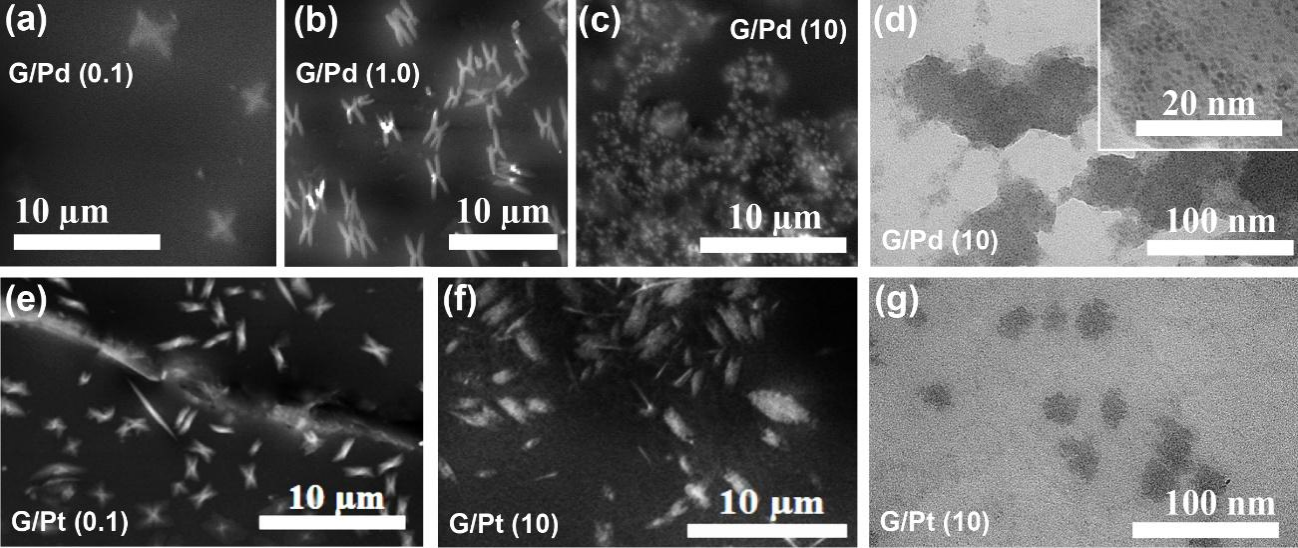
SEM images of G/Pd and G/Pt at different concentrations: (a) G/Pd (0.1 mM), (b) G/Pd (1.0 mM), (c) G/Pd (10 mM), (d) G/Pt (0.1 mM), (e) G/Pt (10 mM); TEM images of (d) G/Pd (10 mM) and (g) G/Pt (10 mM)

Scanning electron microscopy (SEM) images in Figure 5 of G/Pd revealed that palladium clusters tend to assemble into a distinctive chromosome-shaped cluster. At a concentration of 0.1 mM, these Pd clusters exhibit an average diameter of approximately 2 μm. Increasing the Pd concentration reduces the average aggregate size to approximately 0.8 μm, accompanied by a noticeable increase in the number of clusters. Further observation using transmission electron microscopy (TEM) revealed cloud-like clusters with a clear hexagonal geometry, similar to the chromosome-shaped cluster observed in SEM. At higher magnification, individual Pd nanoparticles become discernible within one of these hexagonal cloud clusters. These nanoparticles appear as densely packed dots with an average diameter of about 2.1 nm. A similar morphological trend was observed in the G/Pt nanocomposites.

For instance, at a lower concentration of 0.1 mM, the aggregates also displayed a chromosome-like structure with an average cluster diameter of approximately 2.9 μm. At a higher concentration of 10 mM, the average cluster size decreased to around 2 μm. TEM imaging of these Pt aggregates showed cloud clusters of nanoparticles with an average size of 14 nm.

**Figure 6.**
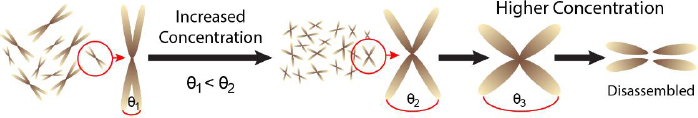
Effects of Pd or Pt nanoparticle precursor concentration on the resulting self-assembly structure



In general, increasing the Pd or Pt precursor concentration shifts the self-assembly process toward smaller metal nanoparticle clusters, as illustrated in Figure 6. The growth rates of both Pd and Pt nanoparticles may be affected by the diffusion of Pd/Pt precursors during hydrothermal synthesis, such that the nanoparticle radius can be approximated by the Ostwald ripening formula typically used for wet chemically synthesized nanoparticles [35-37]:

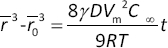

(3)


where 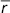 is the average nanoparticle radius at time *t*, and 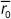 is the average nanoparticle radius at *t* = 0, *γ* / mN m^-1^ is the surface tension at the solid-solution interface, *C*_∞_ is the solubility on a flat surface, *V_m_* is the molar volume, *D* is the diffusion coefficient, *R* is the gas constant, and *T* is the temperature.

To correlate the Pd and Pt particle size distributions shown in the SEM image with the growth process, surface tension was measured using the pendant drop method (see Supplementary material). The surface tension of the G/Pd nanocomposite precursor solution with Pd concentration of 0.1 and 10 mM is found to be 0.11 and 9.7 mN m^-1^, respectively, while the surface tension of the G/Pt nanocomposite precursor solution with Pt concentration of 0.1 mM and 10 mM is found to be 0.37 and 23.94 mN m^-1^, respectively (details in Table S1, Supplementary material). According to Ostwald Ripening theory, the particle radius is proportional to the compound's surface tension and solubility [37]. However, the reduced size of either Pd or Pt in this work is likely not affected by surface tension but rather by the solubility of the precursor in the synthesis of nanocomposites. The amount of PVP plays an important role in the size of the particles formed in the nanocomposite [38,39]. The increase in Pd/Pt concentration from 0.1 to 10 mM during synthesis was accompanied by an increase in PVP concentration. A higher concentration of PVP resulted in lower solubility of metal ions, as the polymer has a higher degree of conformation [40]. Therefore, using a 10 mM concentration of either Pd or Pt yields a smaller particle-size distribution.

**Figure 7.**
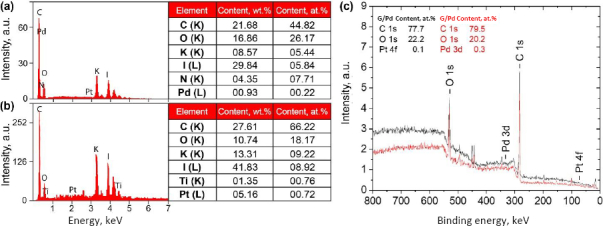
EDX spectra of (a) G/Pd (10) and (b) G/Pt (10) and (c) XPS spectra of G/Pd (10) and G/Pt (10)

Elemental composition analysis is performed by EDX, as shown in Figure 7. The presence of silicon and tin is due to the ITO glass used as the medium during analysis. The EDX result reveals the presence of Platinum, albeit in small amounts, indicating that Pt nanoparticles are successfully synthesized. XPS elemental analysis revealed high intensities of the O 1s and C 1s signals, reflecting the dominant presence of monolayer graphene. The C 1s spectrum displayed a relatively low intensity of hydroxyl functional groups compared to the prominent sp^2^ carbon peak, consistent with a previous report on Pt-modified reduced graphene oxide (rGO) structures [41].

A Pt 4f peak was also detected, though with low intensity, corresponding to a platinum content of approximately 0.1 % by atomic weight. This finding aligns well with the EDX analysis, which showed a Pt atomic percentage of ~0.7 %. Similar observations were made for the G/Pd nanocomposite, where the presence of a Pd-3d peak confirmed the incorporation of palladium. The XPS data indicated a Pd atomic percentage of around 0.3 %, closely matching the ~0.2 % atomic weight measured by EDX, demonstrating consistent results across both characterizations.

### Surface characteristics of G/Pd and G/Pt nanocomposite

**Figure 8.**
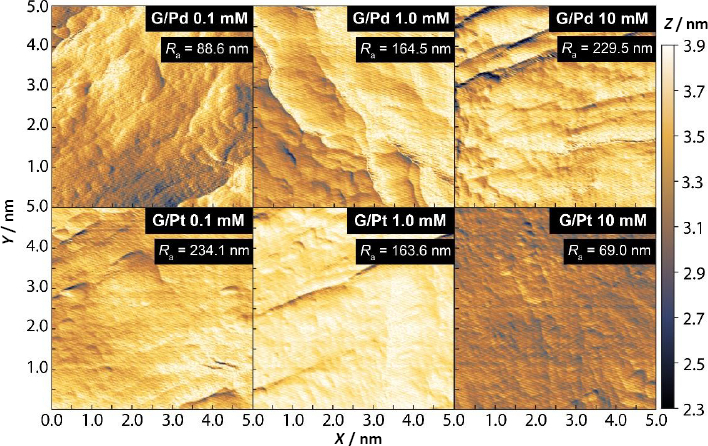
AFM topography image and average roughness (*R*_a_) of G/Pd and G/Pt nanocomposite-modified μPAD surfaces

The surface characteristics of the G/Pd- and G/Pt-modified μPAD were evaluated using atomic force microscopy (AFM) imaging to assess surface roughness and contact angle analysis to measure hydrophobicity. Surface roughness can significantly influence hydrophobicity, with increased roughness typically enhancing hydrophobic behaviour [42]. Figure 8 shows AFM topographic images of μPAD surfaces modified with various concentrations of G/Pd and G/Pt nanocomposites.

The G/Pd-modified surfaces exhibit increasingly uneven textures as the nanocomposite concentration increases, as indicated by more pronounced surface features and higher average roughness values. In contrast, the G/Pt-modified surfaces become smoother as the concentration increases, as reflected by a reduction in surface protrusions and lower average roughness. This suggests that G/Pd deposition increases surface roughness with concentration, whereas G/Pt deposition yields a smoother surface as concentration increases. The observed changes confirm that the nanocomposite composition significantly influences the physical surface properties of the μPAD detection zone. A higher Pd content correlates with increased roughness, whereas higher Pt content leads to a smoother surface.

**Figure 9.**
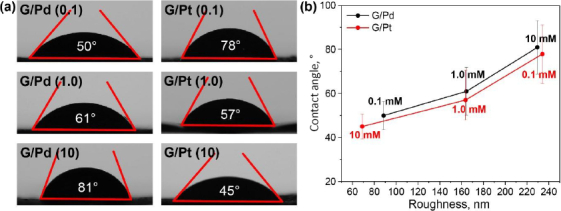
(a) Contact angles of water droplet on G/Pd and G/Pt nanocomposite-modified μPAD surface and (b) correlation between contact angle and surface roughness, with opposite trends observed between G/Pd and G/Pt nanocomposites

To assess the impact of surface modification on fluid flow, contact angle measurements were performed to evaluate the hydrophilicity or hydrophobicity of the modified μPAD surfaces. As shown in Figure 9(a), the contact angle values for all concentrations remained below 90°, indicating that the surfaces remained hydrophilic even after nanocomposite deposition. Figure 9(b) illustrates a linear correlation between contact angle and surface roughness: surfaces with higher roughness tended to exhibit higher contact angles, making them more hydrophobic. This trend is evident in the G/Pd series, where increasing concentration leads to higher surface roughness and, correspondingly, higher contact angles, suggesting a potential drawback for μPAD applications that rely on unobstructed fluid flow. In contrast, G/Pt-modified surfaces become more hydrophilic as the concentration increases, due to reduced surface roughness, thereby promoting better fluid distribution across the detection zone. The enhanced hydrophilicity observed in G/Pt-coated μPADs suggests that fluid flow, whether analytes or reagents, can proceed more smoothly across the detection zone, minimizing the risk of uneven distribution or flow interruption. This characteristic is particularly beneficial for maintaining the performance and reliability of paper-based microfluidic devices.

### Catalytic activity of G/Pd and G/Pt nanocomposite

**Figure 10.**
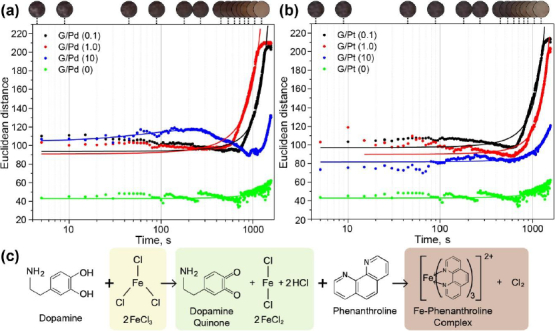
Dynamic response evaluation of dopamine detection using (a) G/Pd and (b) G/Pt nanocomposite as catalyst following the (c) reaction mechanism

The colour change produced by colorimetric reactions with dopamine and FeCl_3_/phenanthroline occurs exponentially over time, with a single peak of maximal intensity, as shown in Figure 10(a). During the addition of FeCl_3_, dopamine reduces Fe^3+^ into Fe^2+^, yielding a colour change from yellow to green. Subsequently, phenanthroline is added to form the Fe-phenanthroline complex, resulting in a colour range from pink to reddish-orange [43]. Comparing the reactions without the addition of a catalyst, the addition of G/Pd and G/Pt significantly enhances colour intensity. However, G/Pd exhibits inconsistent intensity values across different concentrations, showing no clear correlation between concentration and colour intensity. In contrast, G/Pt demonstrates a decrease in colour intensity as its concentration increases. In addition, both catalysts accelerate the reaction as indicated by the reduction in the time constant (*τ*) derived from bi-exponential fitting of the time-dependent colour change, which can be seen in Table 2. This bi-exponential process is plausible because the colorimetric change involves a two-step reaction, as illustrated in Figure 10(c), where 1 corresponds to dopamine-mediated Fe^3+^ reduction and 2 s to Fe-phenanthroline complex formation. The shortest reaction times are observed at lower catalyst concentrations, *i.e.* 1.0 M for G/Pd and 0.1 M for G/Pt. Comparing with the fastest obtained time constant (*τ*), the dopamine detection without any catalyst takes at least 2.5 times longer to achieve a similar intensity as that achieved with the modified μPAD.

**Table 2.** Time constants (τ) of dopamine and NADH colorimetric reaction

Analyte	Reagent	Precursor concentration, mM	G/Pd		G/Pt	
			τ_1_ / s	τ_2_ / s	τ_1 /_ s	τ_2 /_ s
Dopamine	FeCl_3_ and phenanthroline	0	541	∞	541	∞
		0.1	207	611	25	469
		1	87	148	348	630
		10	184	592	896	1642
NADH	Resazurin	0	633	-	633	-
		0.1	256	-	392	-
		1	313	-	449	-
		10	646	-	574	-

**Figure 11.**
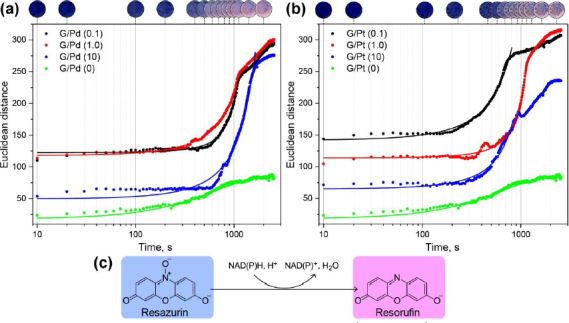
Dynamic response evaluation of NADH detection using (a) G/Pd and (b) G/Pt nanocomposite as catalyst following the (c) reaction mechanism

As with previous detection methods, NADH detection using resazurin also shows an exponential trend over time, as illustrated in Figure 11. NADH was oxidized, releasing a hydrogen ion that reduced the blue-coloured resazurin to resorufin, resulting in a magenta-pink colour [44].

Compared with reactions without a catalyst, the introduction of G/Pd and G/Pt notably increases colour intensity. During NADH detection, G/Pd again shows fluctuating intensity values across different concentrations, whereas G/Pt consistently declines in intensity as concentration increases, mirroring the trend observed in dopamine detection. Both catalysts also accelerate the reaction, consistent with earlier findings, where lower concentrations, 0.1 M for G/Pd and G/Pt, correspond to the shorter τ. For NADH, reactions without a catalyst require approximately twice as long to reach the same intensity as with the modified μPAD. These results from both dopamine and NADH detection highlight the effectiveness of G/Pd and G/Pt in enhancing reaction rates. However, G/Pt appears to be more reliable in producing a consistent trend in colour intensity across concentrations and exhibits slightly shorter τ, *i.e.* faster kinetics, compared to G/Pd.

**Figure 12.**
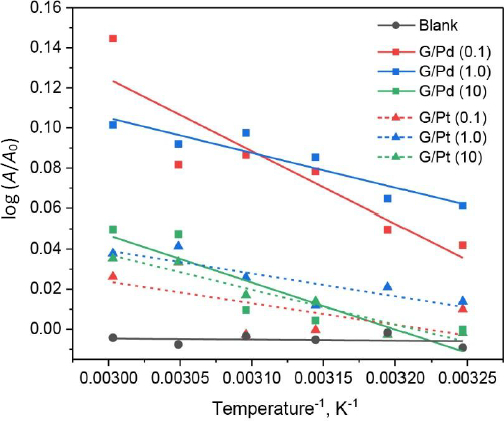
Arrhenius plot of G/Pd derived from absorbance area (at 266.5 / 5 nm) and G/Pt derived from absorbance area (at 209.5 / 5 nm) as colorimetric reaction catalyst

Additional insights into the catalytic performance of G/Pd and G/Pt are assessed by analysing changes in maximum absorbance under varying temperature conditions (see Figure S4 in the Supplementary material). To evaluate the activation energy, the Arrhenius plot derived from temperature-dependent absorbance change is shown in Figure 12. Following the electronic interaction modelled and discussed earlier, the catalytic activity of G/Pd is probed at 266.5 nm, while that of G/Pt is monitored at 209.5 nm.

In general, the absorbance for analyte (*i.e.* dopamine) detection increases with rising temperature irrespective of the catalyst used for the colorimetric reaction. In contrast, reactions without a catalyst exhibit minimal or almost no change in absorbance across the temperature range, resulting in relatively flat curves. Linear regression analysis, summarized in Table 3, reveals that the lowest absorbance-temperature slopes occur at 1.0 M for G/Pd and 0.1 M for G/Pt. These findings align with earlier dynamic response data and further support the catalytic function of G/Pd and G/Pt in lowering the activation energy (*E*_a_) required for the colorimetric reactions. Since the Arrhenius equation relates *E*_a_ to the slope, the observed negative slopes indicate *E*_a_ > 0, which is typical for thermally activated processes.

**Table 3.** Linear regression parameter of Arrhenius plot

Catalyst	Concentration, M	Slope, K	Activation energy, kJ·mol^-1^
G/Pd	0.1	-360.16	2.99
	1.0	-173.68	1.44
	10.0	-234.71	1.95
G/Pt	0.1	-107.53	0.89
	1.0	-113.44	0.94
	10.0	-174.47	1.45

In the present study, the *E*_a_ of G/Pd and G/Pt fall within a narrow range of 0.89 to 2.99 kJ·mol^-1^. There is no clear monotonic trend with increasing Pt or Pd precursor concentration, indicating that different metal nanoparticle loadings do not significantly alter the reaction pathway. Instead, the observed insignificant differences constitute apparent activation energies in typical heterogeneous catalytic systems, where multiple surface processes and site heterogeneity might contribute to the overall kinetics. This consistently small activation energy also suggests that the colorimetric reaction proceeded *via* a surface-mediated mechanism with low intrinsic energy barriers, and hence the subtle differences in surface energetics arise from changes in nanoparticle configuration and metal-graphene interactions.

### Dopamine and NADH detection

In the static response evaluation for neurotransmitter detection using G/Pd and G/Pt catalysts, each catalyst exhibits distinct behaviours as reflected in the calibration curves. Interestingly, both dopamine and NADH detection indicate two linear ranges, *i.e.* the analyte concentration ranges between 0.1 to 1.0 M and 0.5 to 10 mM (see Figure S2 and Figure S3 in the Supplementary material). The use of G/Pd catalyst shows varying performance on dopamine detection at different concentration levels, as seen in Table 4. For detection at low concentrations, G/Pd (0.1) yields the best results with *R*^2^ of 0.83 and LOD of 0.25 μM, while at higher concentrations, G/Pd (1.0) demonstrates a better detection performance with *R*^2^ of 0.94 and LOD of 0.78 mM. In contrast, G/Pt shows better consistency, with G/Pt (10) giving the best results at both concentration levels, with *R*^2^ and LOD of 0.91 and 0.16 μM at lower concentration, and 0.97 and 1.13 mM at higher concentration. Interestingly, G/Pt (1.0), which showed the best dynamic response in earlier tests, had the weakest static detection performance.

**Table 4.** Catalyst performance on dopamine detection

Analyte range	Parameter	G/Pd			G/Pt		
		(0.1)	(1.0)	(10)	(0.1)	(1.0)	(10)
0.1 to 1.0 μM	*R* ^2^	**0.832**	0.759	0.790	0.852	0.753	**0.910**
	Sensitivity, μM^-1^	7.256	**16.554**	16.029	20.955	**28.407**	23.397
	LOD, μM	**0.254**	0.334	0.409	0.297	0.357	**0.164**
	LOQ, μM	**0.771**	1.012	1.240	0.900	1.083	**0.498**
0.5 to 10.0 mM	*R* ^2^	0.614	**0.943**	0.245	0.369	0.082	**0.967**
	Sensitivity, μM^-1^	**1.487**	0.353	0.774	0.461	0.295	**1.293**
	LOD, μM	5.549	**0.782**	12.381	10.632	4.094	**1.128**
	LOQ, μM	16.817	**2.371**	37.517	32.219	12.408	**3.418**

The G/Pd catalyst also exhibits varying performance in NADH detection as seen in Table 5. At higher NADH concentrations, G/Pd (1.0) still shows better detection performance, with an *R*^2^ of 0.98 and an LOD of 0.66 mM. However, at lower concentrations, G/Pd (10) instead exhibited better performance with *R*^2^ of 0.91 and LOD of 0.22 μM. This variability in performance, observed across both dopamine and NADH detection, highlights the inconsistency of G/Pd at low neurotransmitter concentrations. In contrast, G/Pt demonstrated more consistent and reliable detection across all concentration ranges. The G/Pt (10) consistently yields the best result for both neurotransmitters. At low NADH concentrations, G/Pt (10) achieves the best performance with *R*^2^ of 0.914 and LOD of 0.195 μM, while at higher concentrations, it maintains excellent performance with *R*^2^ of 0.98 and LOD of 0.50 mM. Although the difference in detection performance between G/Pt (10) and G/Pt (1.0) is less significant in NADH detection compared to dopamine, G/Pt (1.0) still underperforms relative to G/Pt (10) in static response evaluations.

**Table 5.** Catalyst performance on NADH detection

Analyte Range	Parameter	G/Pd			G/Pt		
		(0.1)	(1.0)	(10)	(0.1)	(1.0)	(10)
0.1 to 1.0 μM	*R* ^2^	0.84	0.19	**0.91**	0.59	0.72	**0.91**
	Sensitivity, μM^-1^	17.21	4.90	**30.83**	4.24	10.79	**28.71**
	LOD, μM	0.27	1.14	**0.22**	0.54	0.37	**0.20**
	LOQ, μM	0.83	3.45	**0.67**	1.65	1.12	**0.59**
0.5 to 10.0 mM	*R* ^2^	0.87	**0.98**	0.97	0.85	0.92	**0.98**
	Sensitivity, μM^-1^	6.78	**7.93**	7.19	6.60	3.44	**8.20**
	LOD, μM	1.26	**0.66**	1.67	1.88	1.22	**0.51**
	LOQ, μM	3.80	**2.00**	5.05	5.70	3.69	**1.53**

Overall, G/Pt catalyst results in a better and more consistent performance in static response, particularly with G/Pt (10), which outperforms other catalyst concentrations in detection parameters of *R*^2^, sensitivity, LOD and LOQ for both dopamine and NADH. Compared to G/Pd, G/Pt shows notably better detection capability, especially with higher *R*^2^, indicating greater linearity and reliability in quantitative measurements. Additionally, the previously identified Pd_Top and Pt_Top configurations, which have been shown to shorten reaction time in dynamic tests, appear to have limited influence on static detection accuracy. While these configurations accelerate the reaction, they may not ensure a complete reaction process within the measurement time and therefore compromise detection precision and performance.

### Structure-properties relationship of G/Pd and G/Pt nanocatalysts

In this work, both G/Pd and G/Pt nanocomposite structures exhibit complex reaction kinetics and sensing performance. As deduced experimentally, all samples exhibit similar *E*_a_, but their sensing performance differs significantly, indicating that the intrinsic reaction energy is not the primary parameter controlling the analytical response. Micromorphology analysis based on SEM observations reveals a morphological evolution in which the nanocomposites form compact chromosome-like structures at lower precursor concentrations, whereas these self-assembled structures break into smaller petal-like assemblies. This morphological transformation increases surface area and improves accessibility of active sites, which is essential as a nanocatalyst in the μPAD platform, where mass transport is limited by capillary flow.

Experimental and DFT-calculated UV-visible absorption spectra for both nanocomposites further confirm that precursor concentration also controls the electronic structure: different precursor concentrations yield different adsorption configurations (Top *vs.* Top2), which in turn affect charge-transfer behaviour. The Top configuration promotes localized charge transfer at the metal-graphene interface, enhancing intrinsic catalytic activity at individual active sites. In contrast, Top2 configurations provide an extended coordination environment, enabling electron delocalization across the metal cluster and the graphene matrix. Therefore, Pd or Pt precursor concentration simultaneously tunes both morphology and electronic properties of the catalyst.

The Arrhenius analysis reveals consistently small activation energies irrespective of G/Pd and G/Pt nanocatalyst composition, suggesting a similar surface-mediated reaction pathway with low intrinsic barriers. Although the trend of *E*_a_ and reaction rate found for G/Pd and G/Pt is somewhat similar, the variations in reaction rate are likely more affected by extrinsic factors, *i.e.* surface accessibility and local environment, rather than changes in Ea. A key finding of this study is that faster kinetics do not directly translate to better sensing performance. Nanocatalysts with hierarchical structure, for example, petal-like assemblies observed at higher concentrations (G/Pt 10 mM), favor efficient intrinsic catalytic activity and exhibit superior sensing characteristics, as reflected in higher linearity and lower detection limits. This discrepancy arises because colorimetric sensing depends not only on reaction rate but also on signal amplification, uniform colour development, and electron-transfer efficiency in the redox process. Overall, the results demonstrate that optimal sensing performance should be balanced between structure and electronic properties. In this system, improving surface accessibility and electron transport is more critical than minimizing *E*_a_.

## Conclusions

This study developed and compared graphene-supported palladium (G/Pd) and platinum (G/Pt) nanocomposite-modified μPADs for the colorimetric detection of dopamine and NADH. Hydrothermal synthesis produced nanocomposites with similar physicochemical characteristics, in which the breaking of C-H bonds on graphene enabled bonding to Pd or Pt nanoparticles. While G/Pd surfaces became rougher and more hydrophobic, G/Pt surfaces were smoother and more hydrophilic, beneficial for fluid flow on paper-based platforms. Structural modelling showed that nanoparticles grow atomically on graphene, with Pd_Top and Pt_Top configurations correlating to optimal catalytic behaviour. In dopamine detection, G/Pd exhibits varying best detection performance at specific concentrations. G/Pt, particularly G/Pt (10), offers greater consistency, with high R^2^ and low LOD across wide concentration ranges. Similar trends are observed for NADH, where G/Pt again demonstrated better, more stable performance than the variable results from G/Pd. Although Pd_Top and Pt_Top configurations improved dynamic response, it should be noted that the kinetic parameters and sensing performance follow different structure-activity relationships, where catalysts with comparable or even slower intrinsic kinetics can exhibit superior analytical sensitivity due to enhanced morphology and interfacial properties. These findings underline the importance of process optimization during nanocomposite synthesis. Future work should investigate the long-term stability of G/Pt nanocomposites and explore multiplex detection capabilities. Overall, this work demonstrates the potential of G/Pd and G/Pt-modified μPADs as an accessible and reliable point-of-care biosensing platform for neurotransmitter detection.

## Supplementary material

Additional data are available at https://pub.iapchem.org/ojs/index.php/admet/article/view/3247, or from the corresponding author on request.


