# Supplementary material for: Graphene-supported Pd/Pt nano-catalysts for enhanced colorimetric detection of dopamine and NADH using paper-based microfluidic devices
Source: ADMET DMPK. 2026 Apr 7;14:3247. doi: 10.5599/admet.3247 (PMC13147513; doi:10.5599/admet.3247)
Supplement: Supplementary file 1 [file ADMET-14-3247-S1.pdf]

Open Access : ISSN : 1848-7718
http://www.pub.iapchem.org/ojs/index.php/admet

Supplementary material to

Graphene-supported Pd/Pt nano-catalysts for enhanced colorimetric detection of dopamine and NADH using paper-based microfluidic devices

Ruri Agung Wahyuono[1,2,*], Jovin Jovin[1], Ignacius Gilbert Chano[1], Arda Fridua Putra[1], Annisa Septyana Ningrum[1], Muhammad Yusuf Hakim Widianto[3], Irkham Irkham[4], Yeni Wahyuni Hartati[4], Wulan Tri Wahyuni[5], Isnaini Rahmawati[6], Chi-Hsien Huang[1,7] and Yi-Ting Lai[7]

[1]*Department of Engineering Physics, Institut Teknologi Sepuluh Nopember, Surabaya 60111, Indonesia*
[2]*School of Interdisciplinary Management and Technology, Institut Teknologi Sepuluh Nopember, Surabaya 60264, Indonesia*
[3]*Department of Mathematics, Institut Teknologi Sepuluh Nopember, Surabaya 60111, Indonesia*
[4]*Department of Chemistry, University of Padjadjaran, Sumedang 45363, Indonesia*
[5]*Department of Chemistry, Institut Pertanian Bogor (IPB) University, Bogor 16680, Indonesia*
[6]*Department of Chemistry, University of Indonesia, Depok 16424, Indonesia*
[7]*Department of Materials Engineering, Ming Chi University of Technology, New Taipei City 243303, Taiwan-*

ADMET & DMPK **14** (2026) 3247; https://doi.org/10.5599/admet.3247

Pendant drop test

Pendant drop test was carried out by measuring the diameter of fluid right before it drops from a capillary tube. The diameter measured to calculate the surface tension is the maximum droplet diameter of (D_e) and the diameter of the fluid right before it drops (D_s) as illustrated in Figure S1.

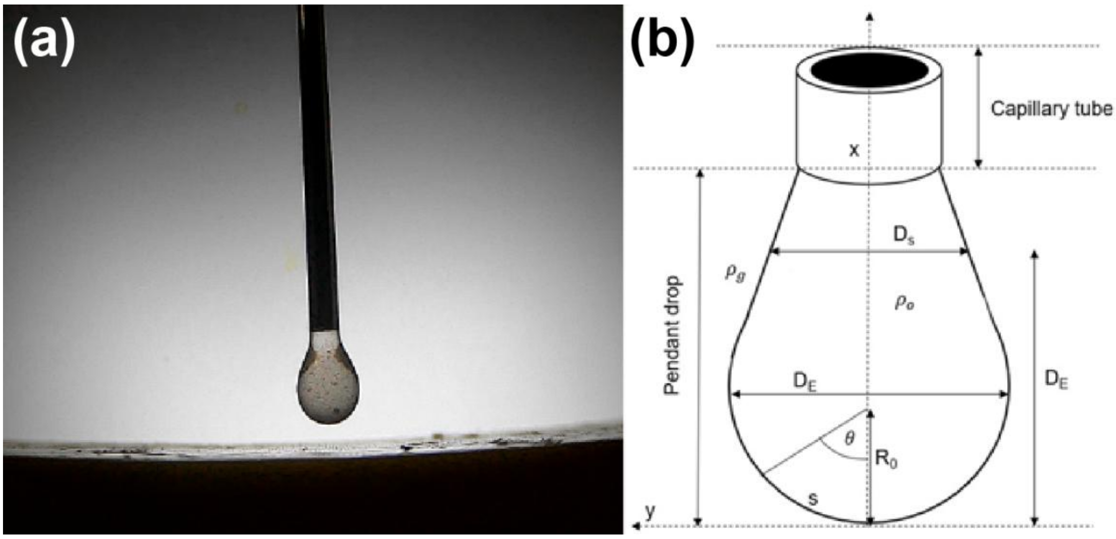

Fgure S1. (a) Pendant drop test with the (b) geometrical parameters

The surface tension was then calculated using Equation (S1):

$$\gamma = \frac{\Delta \rho g D_E^{\,2}}{\beta} \qquad \text{(S1)}$$

where γ / mN·m^{-1} is the surface tension, $\Delta\rho$ / g·cm^{-3}is the density difference between fluid and air, D_E / cm is maximum droplet diameter as illustrated in Figure S1(b). The β is the shape factor of pendant drop expressed by Young-Laplace in the first order 3 dimensionless Equation (S2):

$$\beta = 0.1284\text{-}0.7577\frac{D_S}{D_E} + 1.7713\left(\frac{D_S}{D_E}\right)^2 \text{-}0.5426\left(\frac{D_S}{D_E}\right)^3 \quad \text{(S2)}$$

where D_S / cm is the necking droplet diameter. The calculated surface tension of G/Pt and G/Pd nanocomposite precursor solution is summarized in Table S1.

Tabel S1. Surface tension of various nanocomposite variations

Precursor concentration, mM	Surface tension, mN m^{-1}	
	G/Pd	G/Pt
0.1	0.11	0.37
1.0	1.17	3.44
10.0	9.70	23.94

Calibration curve

As described in the main manuscript, both dopamine and NADH detection indicate two linear range, *i.e.*, the analyte concentration ranges between 0.1 to 1.0 μM and 0.5 to 10 mM as depicted in Figure S2 and Figure S3, respectively. These calibration curves were used to evaluate sensing performance evaluated in the main manuscript.

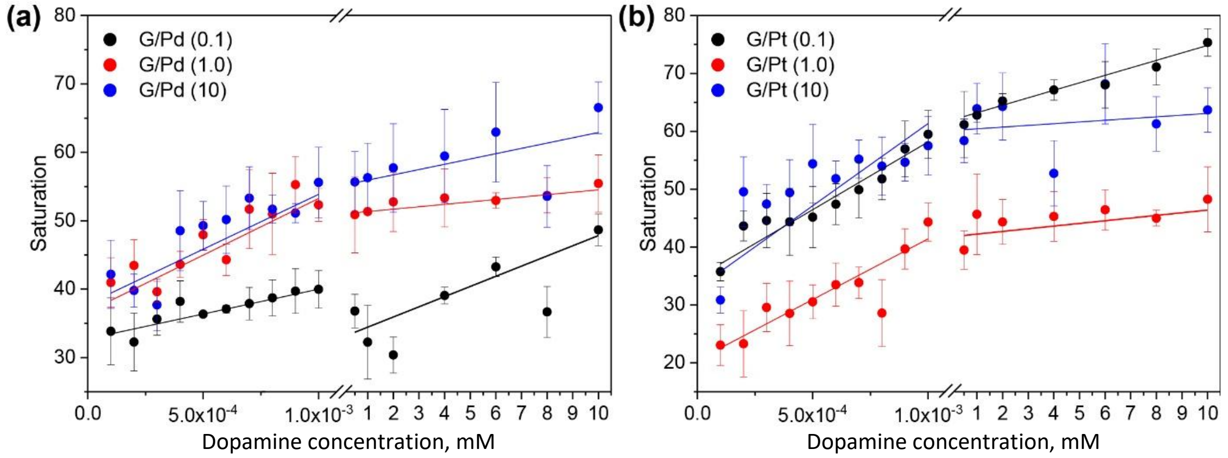

Figure S2. Calibration curve of static response evaluation on dopamine detection using (a) G/Pd and (b) G/Pt nanocomposite as catalyst

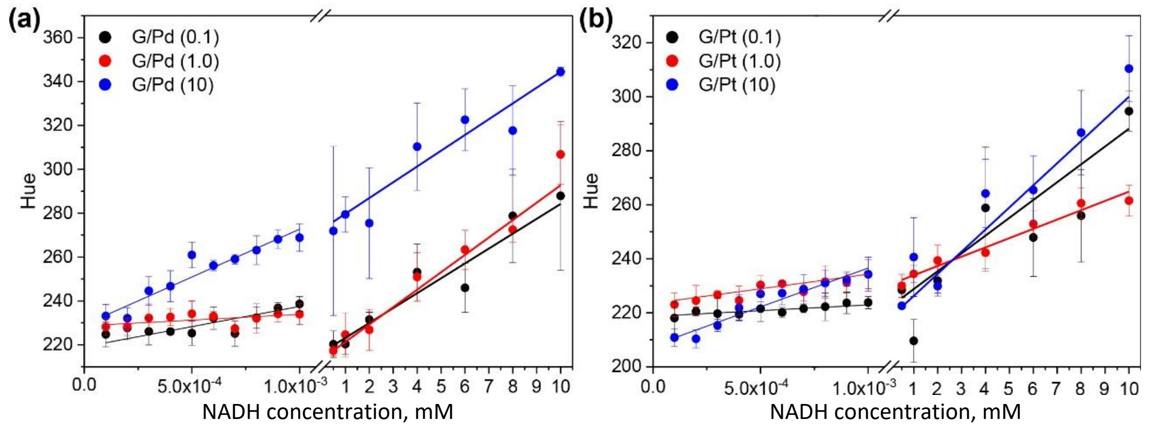

Figure S3. Calibration curve of static response evaluation on NADH detection using (a) G/Pd and (b) G/Pt nanocomposite as catalyst

CC BY

Temperature-dependent UV-Vis spectra

To characterize the activation energy of G/Pd and G/Pt catalyst, temperature dependent absorption spectra of dopamine using different catalyst were recorded and shown in Figure S4.

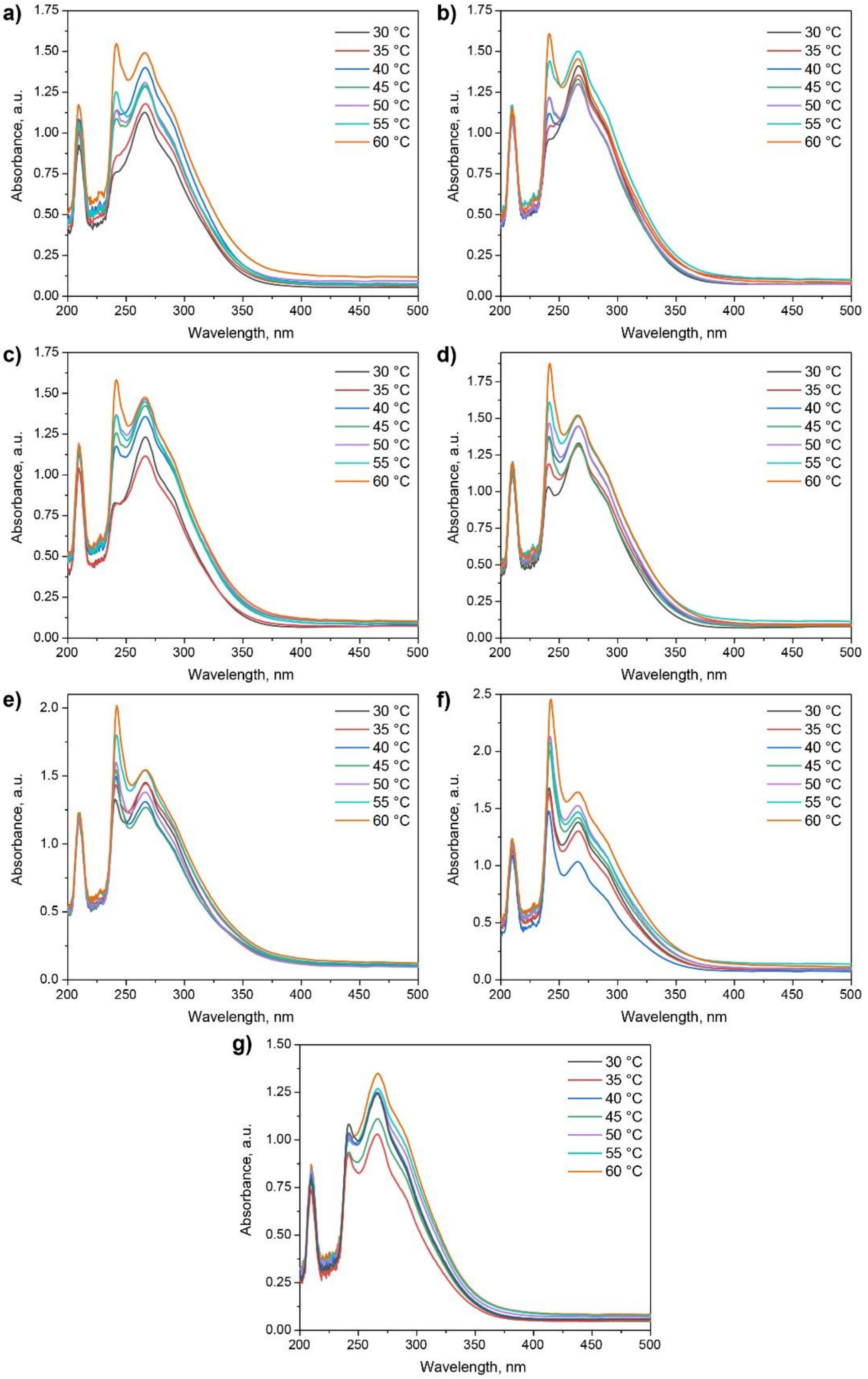

Figure S4. UV-vis absorbance spectra of dopamine reaction with (a) G/Pd (0.1), (c) G/Pd (1.0), (e) G/Pd (10), (b) G/Pt (0.1), (d) G/Pt (1.0), (f) G/Pt (10) and (g) without catalyst at varying temperature.

The activation energy is derived from the slope of log (A/A_0) *vs.* $1/T$ depicted in Figure S5. The Arrhenius plot is built from the absorbance change at the wavelength of 209.5, 241.5 and 266.5 nm which are indicative of metal nanoparticle interactions in graphene matrix. The activation energy from all wavelengths is summarized in Table S2.

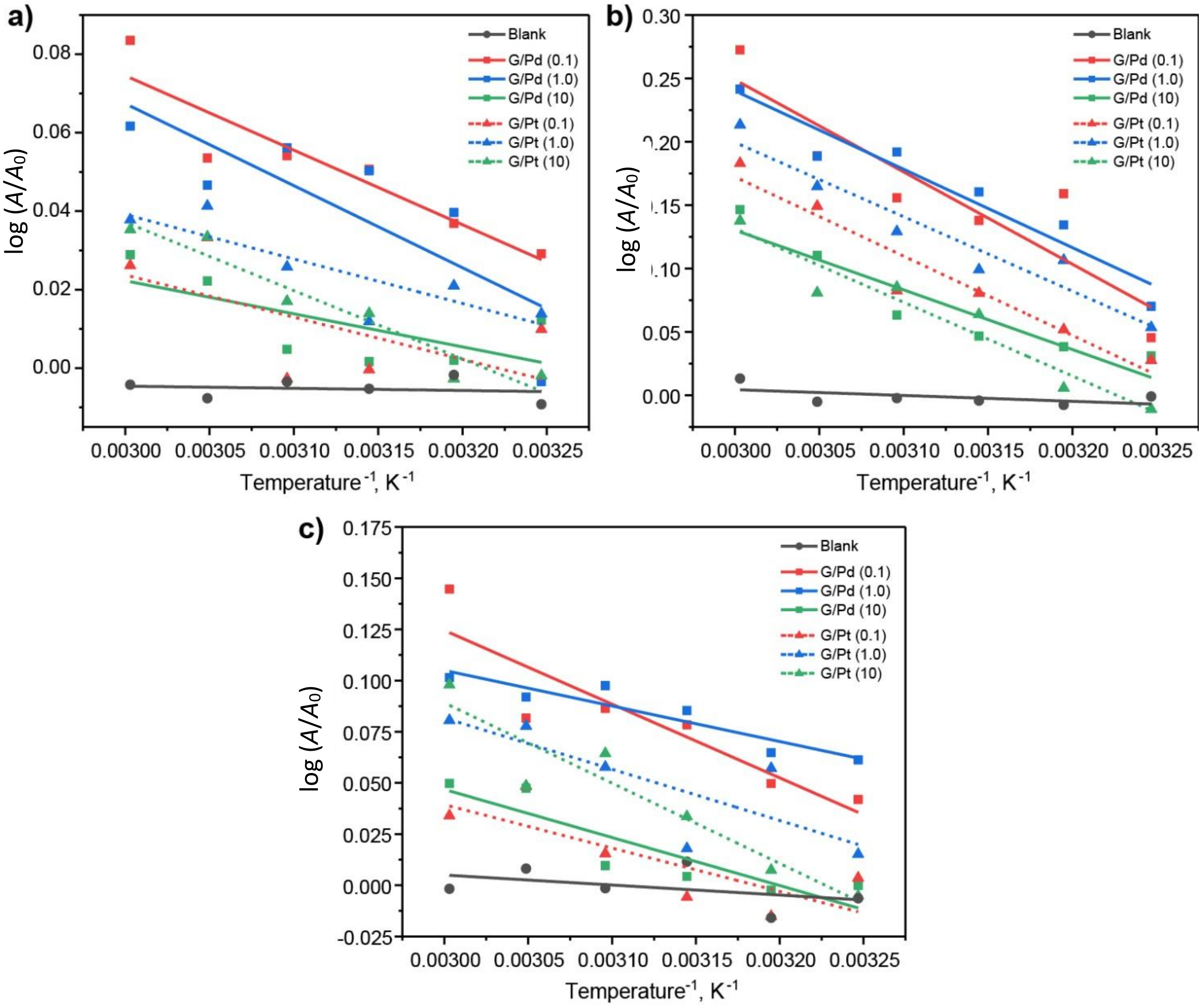

Figure S5. Arrhenius plot at (a) 209.5, (b) 241.5 and (c) 266.5 nm of G/Pd and G/Pt as colorimetric reaction catalyst

Table S2. Linear regression parameter of Arrhenius plot

Catalyst	Concentration of Pt or Pd, M	Slope, K			E_a / kJ·mol^{-1}		
		209.5 nm	241.5 nm	266.5 nm	209.5 nm	241.5 nm	266.5 nm
G/Pd	0.1	-190.06	-727.54	-360.16	1.58	6.05	2.99
	1.0	-209.24	-617.99	-173.68	1.74	5.14	1.44
	10.0	-84.35	-470.76	-234.71	0.70	3.91	1.95
G/Pt	0.1	-107.53	-625.30	-211.88	0.89	5.20	1.76
	1.0	-113.44	-586.64	-250.50	0.94	4.88	2.08
	10.0	-174.47	-580.94	-392.38	1.45	4.83	3.26

CC BY
